# Molecular identification of rhamnolipids produced by *Pseudomonas oryzihabitans* during biodegradation of crude oil

**DOI:** 10.3389/fmicb.2024.1459112

**Published:** 2024-08-20

**Authors:** Saman Hosseini, Rouhallah Sharifi, Alireza Habibi, Qurban Ali

**Affiliations:** ^1^Department of Plant Protection, College of Agriculture and Natural Resources, Razi University, Kermanshah, Iran; ^2^Faculty of Petroleum and Chemical Engineering, Razi University, Kermanshah, Iran; ^3^Department of Biology, College of Science, United Arab Emirates University, Al-Ain, United Arab Emirates

**Keywords:** bioremediation, biosurfactant production, critical micelle concentration, emulsification capacity, hydrocarbon-degrading bacteria, rhamnolipid

## Abstract

**Introduction:**

The ability to produce biosurfactants plays a meaningful role in the bioavailability of crude oil hydrocarbons and the bioremediation efficiency of crude oil-degrading bacteria. This study aimed to characterize the produced biosurfactants by *Pseudomonas oryzihabitans* during the biodegradation of crude oil hydrocarbons.

**Methods:**

The biosurfactants were isolated and then characterized by Fourier transform infrared (FTIR), liquid chromatography-mass-spectrometry (LC–MS), and nuclear magnetic resonance spectroscopy (NMR) analyses.

**Results:**

The FTIR results revealed the existence of hydroxyl, carboxyl, and methoxyl groups in the isolated biosurfactants. Also, the LC–MS analysis demonstrated a main di-rhamnolipid (l-rhamnopyranosyll-rhamnopyranosyl-3-hydroxydecanoyl-3-hydroxydecanoate, Rha-Rha-C10-C10) along with a mono-rhamnolipid (l-rhamnopyranosyl-b-hydroxydecanoylb-hydroxydecanoate, Rha-C10-C10). In agreement with these findings, the NMR analysis confirmed the aromatic, carboxylic, methyl, sulfate moieties, and hexose sugar in the biosurfactants. The emulsion capacity of the biosurfactants decreased the surface tension of the aqueous system from 73.4 mN m^−1^ to around 33 mN m^−1^ at 200 mg L^−1^ as the critical micelle concentration. The emulsification capacity of the biosurfactants in the formation of a stable microemulsion for the diesel-water system at a wide range of pH (2–12), temperature (0–80°C), and salinity (2–20 g L^−1^ of NaCl) showed their potential use in oil recovery and bioremediation through the use of microbial enhancement.

**Discussion:**

This work showed the ability of *Pseudomonas oryzihabitans* NC392 cells to produce rhamnolipid molecules during the biodegradation process of crude oil hydrocarbons. These biosurfactants have potential in bioremediation studies as eco-friendly and biodegradable products, and their stability makes them optimal for areas with extreme conditions.

## 1 Introduction

Petroleum hydrocarbons are the widespread organic contaminants in soil and water, which pose many environmental concerns (Inieke et al., 2018). Recent studies have concentrated on finding fast, safe, and cost-effective approaches to remove and remediate these contaminations (Mille et al., 2006). Among the remediation methods of petroleum contaminations, bioremediation using degrading microorganisms as a cost-effective and environmentally friendly method is often the most appropriate method to maintain environmental balance (Mulligan, 2009). One of the most critical advantages of microorganisms in degrading petroleum hydrocarbons is their capability to emulsify hydrocarbons in solution by *in-situ* synthesizing biosurfactants, which cause to facilitate the accessibility of hydrocarbons in aqueous solutions and subsequently more degrading by the enzymatic systems (Adebusoye et al., 2008; Ibrahim et al., 2013). Some experiments showed the application of the *in-situ* biosurfactant-producing cells in the degradation of hydrocarbons had environmental and technical benefits in comparison with the addition of chemical surfactants (Shan et al., 2016; Sun et al., 2021; Sajadi Bami et al., 2022).

Biosurfactants are compounds that have both lipophilic and hydrophilic properties. This amphiphilic structure allows them to reduce the surface tension in the mixture of water and hydrocarbon (Santos et al., 2016). Bacteria are among the agents capable of biosurfactant synthesis (Marchant and Banat, 2012). The chemical composition and microbial origin of biosurfactants determine their classification, unlike chemical surfactants that are classified based on their polar group (Sajadi Bami et al., 2022). Typically, they are made up of a hydrophilic part with amino acids, polysaccharides, and electrically charged particles and a hydrophobic part with saturated or unsaturated fatty acids (Hajfarajollah et al., 2018; Abo Elsoud, 2021). Based on their molecular structure, biosurfactants are classified into different groups, including phospholipids, glycolipids, lipopeptides, neutral lipids, lipopolysaccharide-protein complexes, and short and long-chain fatty acids (Roy, 2017; Sánchez, 2022). Biosurfactants can tolerate salt concentrations, high temperatures, and extreme environmental conditions and remain stable, which in turn can facilitate the bioremediation process in environments with unfavorable ecological conditions (Joe et al., 2019; Deng et al., 2020; Ravindran et al., 2020). Generally, organic contaminations can be degraded using biosurfactants as a low-cost technique without requiring specialized equipment. These compounds lead to removing the contamination by emulsifying the hydrophobic contamination and increasing the access of the degrading bacteria to the contamination without producing a toxic product (Effendi et al., 2018; Sajadi Bami et al., 2022). Although some biosurfactant production by *Pseudomonas* spp. during bioremediation processes are reported in literature (Patowary et al., 2017; Zdarta et al., 2019; Rehman et al., 2021; Sun et al., 2021; Sajadi Bami et al., 2022; Sánchez, 2022), to the best of our knowledge, this is the first record of high-efficiency biosurfactant production by *P. oryzihabitans*. The previous work showed that *P. oryzihabitans* NC392 has a high ability to rapid degradation of aliphatic hydrocarbons of crude oil contaminants via the terminal oxidation pathway (Hosseini et al., 2024). The objective of this study is to isolate and characterize the surface-active agents produced during this biodegradation process. The emulsification capacities of the biosurfactants was evaluated for the microemulsion formation from the water–oil system, and their stability at different pH, temperature, and salinity was determined.

## 2 Materials and methods

### 2.1 Bacterium strain and cultural conditions

The bacterium strain used in this study was obtained from the bacterial collection of the Department of Plant Protection, Razi University, Kermanshah, Iran, previously isolated by the present research team from petroleum-contaminated soil (Hosseini et al., 2024). Biochemical characteristics and 16SrRNA ribosomal region sequencing showed that the bacterium with accession number OP963714 belongs to *Pseudomonas oryzihabitans* strain NC392. The bacterial strain was cultured on a nutrient agar (NA) medium and incubated for 72 h at 28°C. The bacterium strain was kept at −80°C in a 50% glycerol solution for further study.

### 2.2 Degrading of crude oil and biosurfactant production

To investigate the ability of the strain to remove crude oil and produce biosurfactants, the overnight culture of the bacterium in the M9 mineral medium was used (Cappello et al., 2012). For this purpose, Erlenmeyer flasks containing 100 mL of M9 mineral medium (g L^−1^) (NaCl, 0.5; Urea, 1; Na_2_HPO_4_, 6.78; KH_2_PO_4_, 3; MgSO_4_.7H_2_O, 0.1) to which 15 g L^−1^ of crude oil was added as a carbon source, using 1 v/v% bacterium suspension with a population density of 10^7^–10^8^ colony forming unit (CFU mL^−1^) at 28°C of 130 rpm for 120 h was mixed and incubated. After that, the bacterial cells were precipitated using a centrifuge at 6000 rcf for 10 min, and the cell-free supernatants (CFS) were kept in the refrigerator to examine the biosurfactant. Furthermore, the ability of bacteria to remove crude oil was measured. The CFS was mixed well with an equal volume of n-hexane and vigorously vortexed for 4 min to transfer the remaining crude oil to the organic hexane phase. Then, the absorbance of the organic phase was measured at 420 nm (Rahman et al., 2002). To calibrate, different concentrations (0–15 g L^−1^) of crude oil were prepared in n-hexane. To convert the absorbance values to concentrations, Equation 1 is used as the standard curve.


(1) $$\begin{array}{l} \mathrm{Residual crude oil}\;\\ \mathrm{concentration}\;\left(g\;L^{-1}\right)=6.111\times {OD}_{420\;\mathrm{nm}}-0.9626 \end{array}$$

The crude oil removal efficiency and biodegradation rate were calculated using Equations 2, 3 (Habibi and Babaei, 2017; Sun et al., 2019). In this study, a non-inoculated medium and a medium inoculated with a non-hydrocarbon-degrading bacterium, *Pseudomonas* sp. strain SH1, were considered as abiotic and biotic negative controls, respectively.


(2) $$\mathrm{Crude}\;\mathrm{oil}\;\mathrm{removal}\;\mathrm{efficiency}\;\left(\%\right)=100\times \left(\frac{C_{I}-C_{E}}{C_{I}}\right)$$

(3) $$\mathrm{Biodegradation rate}\;\left(g\;L^{-1}h^{-1}\right)=\frac{\begin{array}{l} \mathrm{Crude oil removal}\;\\ \mathrm{efficiency} \end{array}}{100}\times \frac{C_{I}\;}{\mathrm{Time}\;\left(h\right)}$$

Where *C_I_* and *C_E_* denote the concentrations of crude oil at the beginning and end of the experiment, respectively.

### 2.3 Investigation of potential biosurfactant produced by *Pseudomonas oryzihabitans*

#### 2.3.1 Hemolytic activity

To investigate the hemolytic activity of the *P. oryzihabitans* strain, a pure colony from an overnight bacterial culture was cultured on a blood agar (BA) medium containing 5% defibrinated sheep blood and incubated for 48–72 h at 35°C. Hemolytic activity was evaluated by creating a clear halo around a colony (Mulligan et al., 1984; Uyar and Sağlam, 2021).

#### 2.3.2 Drop collapse test

The drop collapse of crude oil was performed with the Enzyme-linked immunosorbent assay (ELISA) method (Jain et al., 1991; Bodour and Miller-Maier, 1998; Youssef et al., 2004). For this purpose, 10 μL of CFS was added into ELISA wells containing 100 μL of liquid paraffin. A positive result is considered if the added CFS drop moves to the bottom of the well or the CFS spread partially to complete it on the paraffin surface. The results were checked at 1, 30, and 60 s. In the present experiment, the chemical surfactant Tween 20 was used as a positive control, and the M9 mineral medium was used as a negative control.

#### 2.3.3 Oil spreading assay

To investigate the spreading of crude oil, crude oil (100 μL) was added to the center of a Petri dish containing 30 mL of distilled water, and then 100 μL of CFS was placed at the center of the crude oil. The measurement was conducted to determine the diameter of the clear zones. In the present experiment, the chemical surfactant Tween 20 was used as a positive control, and the M9 mineral medium was used as a negative control (Morikawa et al., 2000; Huang et al., 2020).

#### 2.3.4 Emulsification index

For this experiment, 5 mL of CFS was poured into a tube, and 5 mL of diesel was added. The tube was vigorously mixed for 2 min and kept statically at 25°C for 24 h. By dividing the height of the emulsion layer by the height of the total liquid multiplied by 100, the percentage of emulsification was calculated. In the present experiment, the chemical surfactant Tween 20 at 1.095 g mL^−1^ concentration was used as a positive control, and the M9 mineral medium was used as a negative control (Cooper and Goldenberg, 1987; Iqbal et al., 1995; Habibi and Karami Rahimabadi, 2018).

#### 2.3.5 Measurement of surface tension

Using the force tensiometer model TM-TN-555, Nanotos Company, Iran, at 25°C, the surface tension of the CFS was measured. For this experiment, the platinum ring of the device was submerged in 50 mL of CFS, and its surface tension was determined in mN m^−1^ (Iqbal et al., 1995; Hassanshahian, 2014; Nayarisseri et al., 2019).

### 2.4 Determining the critical micelle concentration

First, 1,000 mg L^−1^ of crude biosurfactant was dissolved in distilled water. Then, it was successively diluted (0–500 mg L^−1^), then the emulsification index (E24) and surface tension values of the prepared dilutions were measured, respectively (Abdel-Mawgoud et al., 2008). The lowest value of surface tension and the highest value of emulsification index were considered as the critical micelle concentration.

### 2.5 Biosurfactant isolation

The CFS was separated using a centrifuge at 6,000 rpm for 10 min, then the pH of the CFS was adjusted to 2 using 6 N HCl and kept in the refrigerator at 4°C for 24 h. After that, the CFS was centrifuged at 12,000 rcf for 15 min. Finally, the sediment was washed with 10 mL of methanol and dried in a vacuum oven at 35°C (Habibi and Babaei, 2017).

### 2.6 Evaluation of biosurfactant stability

The emulsification index (E24) was measured in different conditions of temperature, salinity, and pH to determine the stability of biosurfactant. For this purpose, the tubes containing 5 mL of CFS were placed at various temperatures of 0, 20, 40, 60, and 80°C for 30 min, and then they were brought to ambient temperature at room temperature. The pH of the cell-free supernatants were adjusted to 2, 7, and 12 using 2 N HCl and NaOH solutions. Different salinity of 2, 5, 10, 15, and 20% were prepared by adding appropriate amounts of NaCl. At the end of the experiments, the emulsification index was calculated (Obayori et al., 2009). The experiments were performed in three replicates, and the mean values ± the standard errors were reported.

### 2.7 Fourier transform infrared spectroscopy

The FTIR analysis of the biosurfactant was performed on a Bruker ALPHA II FTIR Spectrometer. Approximately 1 mg of extracted biosurfactant was ground with KBr (100 mg) and pressed into a pellet for seconds. The wavelength range of the scanning was 400–4,000 cm^−1^. The IR spectra were analyzed using Bruker OPUS 7.0.129 FTIR (spectroscopy) software (Chandran and Das, 2010).

### 2.8 Nuclear magnetic resonance

To confirm the structure and further characterize the extracted crude biosurfactant, nuclear magnetic resonance (NMR) analysis was performed. Hence, 5 mg of purified biosurfactant was dissolved in 0.5 mL of dimethyl sulfoxide-D6 (DMSO-d6) (99.9%). All ^13^C and ^1^H nuclear magnetic resonance (NMR) spectra were measured at 25°C using a VARIAN - INOVA spectrometer (United States) set at 500 MHz. In parts per million (ppm), the chemical shift was observed (Mohammed et al., 2021; Zargar et al., 2022).

### 2.9 Liquid chromatography-mass spectrometry

An Agilent 1200 series LC system with a thermostat column chamber, quaternary transferring pump, degasser, and Rheodyne 7725i manual injector valve with a 20 μL sample loop (Cotati, CA, United States) was used for the liquid chromatography-mass spectrometry (LC–MS) analysis. Mass analysis was carried out using an Agilent 6410 Triple Quadrupole mass spectrometer (Agilent Technologies, Palo Alto, CA, United States) and the Agilent Mass Hunter Workstation data collection system B.01.03. Electrospray ionization (ESI) in both negative and positive modes at 3,000 V capillary voltage was used for ionization. Nebulizer gas was nitrogen with 40 psi pressure and 100°C source temperature. Nitrogen was utilized as a drying gas by heating it to 300°C and delivering it at a flow rate of 10 L min^−1^. The voltage of the fragmentor was set at 135 V, and the dwell time was 200 ms (Déziel et al., 1999; Babaei and Habibi, 2018).

### 2.10 Statistical analysis

All the tests were performed in a completely random design, and three repetitions were considered for each treatment. Analysis of variance (ANOVA) procedure in SAS (version 9.3) was used to analyze the data. The mean comparison was performed using Duncan’s multiple-range test. GraphPad Prism 8 software was used to draw the graphs.

## 3 Results and discussion

### 3.1 Biodegradation of crude oil hydrocarbons and potential biosurfactant production

**Figure 1:**
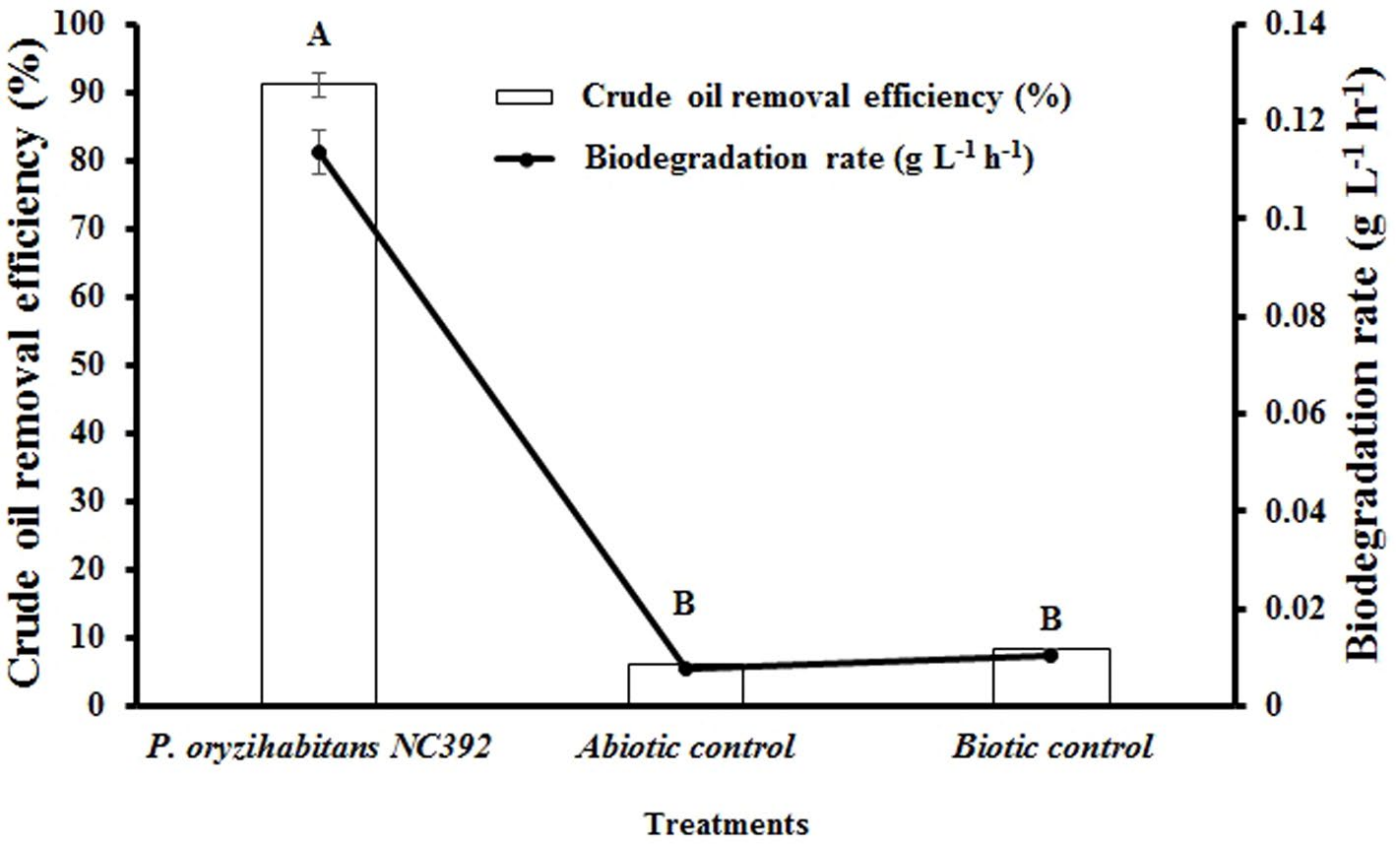
Crude oil removal efficiency and biodegradation rate of crude oil biodegradation by the selected strains during 120 h in M9 medium containing 15 g L^−1^ of crude oil. The non-inoculated medium was abiotic control and a non-crude oil-degrading bacterium *Pseudomonas* sp. SH1 was a negative biotic control. Bars on the columns indicate the standard error of the mean. The difference between means with common letters is not statistically significant.

The biodegradation of crude oil hydrocarbons from aqueous environment was evaluated by *P. oryzihabitans* at an initial concentration of 15 g L^−1^. The results of these experiments showed that crude oil hydrocarbons could be efficiently removed after 120 h with a removal efficiency of 91% at a biodegradation rate of 0.11 g L^−1^ h^−1^ (Figure 1). This indicates the high ability of *P. oryzihabitans* to use hydrocarbons of crude oil as the source of carbon. In previous studies, the ability of *P. oryzihabitans* to degrade various compounds has been proven (Song, 2009; Aresta et al., 2010). The high removal efficiency of *P. oryzihabitans* to degrade crude oil can be due to the production of biosurfactant by *P. oryzihabitans*, followed by increasing the access of *P. oryzihabitans* to crude oil in the medium and using it as a carbon source. While *Pseudomonas* sp. strain SH1 (a non-hydrocarbon-degrading bacterium) was used as a negative control, the result demonstrated that this strain could not remove crude oil hydrocarbons from the medium. *Pseudomonas* sp. strain SH1 is a plant pathogen adapted to plant surfaces. Also, the abiotic control test result showed that the removal of hydrocarbons due to adsorption and ventilation was negligible during the experiments. So, it is expected that *P. oryzihabitans* improves the bioavailability of insoluble hydrocarbons in water with an *in-situ* production of surface active agents, which increases the bioremediation performance of the cells in removal of hydrocarbon contaminants (Ron and Rosenberg, 2002; Batista et al., 2006).

The isolation process of the biosurfactants from *P. oryzihabitans* culture obtained 4 g L^−1^ of dried powder of biosurfactant. To evaluate the emulsification ability of the biosurfactant, rapid screening methods, including the bacterial hemolytic activity, oil drop collapse test, oil spreading, emulsification index (E24), and surface tension measurements were performed on the CFS. The results of Table 1 revealed that *P. oryzihabitans* had hemolytic activity of the beta hemolysis type in the BA medium. This potential has been previously considered as an indicator for biosurfactant production by microorganisms (Mulligan et al., 1984; Ibrahim et al., 2013). CFS was able to disperse crude oil with a diameter of 38 mm on the surface of distilled water, which was comparable to the dispersion of crude oil with a diameter of 47 mm by the Tween 20 as positive control. Furthermore, CFS showed a positive result in the oil drop collapse test and was able to settle in less than 60 s. These two tests are important primary screening tests for bacterial isolates for biosurfactant production (Youssef et al., 2004). Also, the emulsifying index (E24) shows the emulsion height relative to the total height of the mixture of CFS and diesel (Peele et al., 2016). The result of the experiment showed that the microemulsion layer made from the supernatant phase and diesel was about 52% of the total mixture. The positive control in the presence of Tween 20 (chemical surfactant) showed 50% emulsification index at 1.095 g mL^−1^. This amount of emulsification shows the high potential of *P. oryzihabitans* to produce surface active agents in the culture during the biodegradation process. In fact, high emulsification index has been considered as the main factor for the identification of potential biosurfactant-producing bacteria (Rahman et al., 2003; Uyar and Sağlam, 2021). According to earlier studies, the surface tension of water in the equilibrium state is approximately 70 mN m^−1^, and biosurfactants can reduce this value due to their surface activities (Khopade et al., 2012b; Hauner et al., 2017). In this study, *P. oryzihabitans* was able to reduce the surface tension of the M9 medium from 68 to 31 mN m^−1^, which is mainly due to the presence of biosurfactants, which will be able to transfer the crude oil layer formed on the surface of the medium to the liquid phase by reducing the surface tension. This level of reduction in surface tension indicates the high ability of *P. oryzihabitans*, considering that in previous studies, the reduction of surface tension below 40 mN m^−1^ was introduced as a good surface active agent (Walter et al., 2010). The overall summary from these investigations confirms the potential biosurfactant production by *P. oryzihabitans* during the biodegradation process. Since all preliminary investigation experiments were performed using the cell-free supernatant, it can be concluded that bacterial cells produce biosurfactants and secrete them into the extracellular space (Batista et al., 2006; Hassanshahian, 2014).

**Table 1:** The emulsification capacities of the cell-free supernatant (CFS) obtained from the biodegradation process by *P. oryzihabitans* NC392.

Aqueous phase system	Oil spreading (mm) ± SD	Drop collapse	Hemolytic activity	Emulsification index (%) ± SD	Surface tension (mN m^−1^) ± SD
CFS of *P. oryzihabitans* NC392	38 ± 0.76	+	+	52 ± 1.04	33 ± 1.15
Tween 20 (1.095 g mL^−1^)	47 ± 0.00	++	ND	50 ± 0.00	ND
Distilled water (M9 medium)	0	−	ND	0	68 ± 0.00

**Figure 2:**
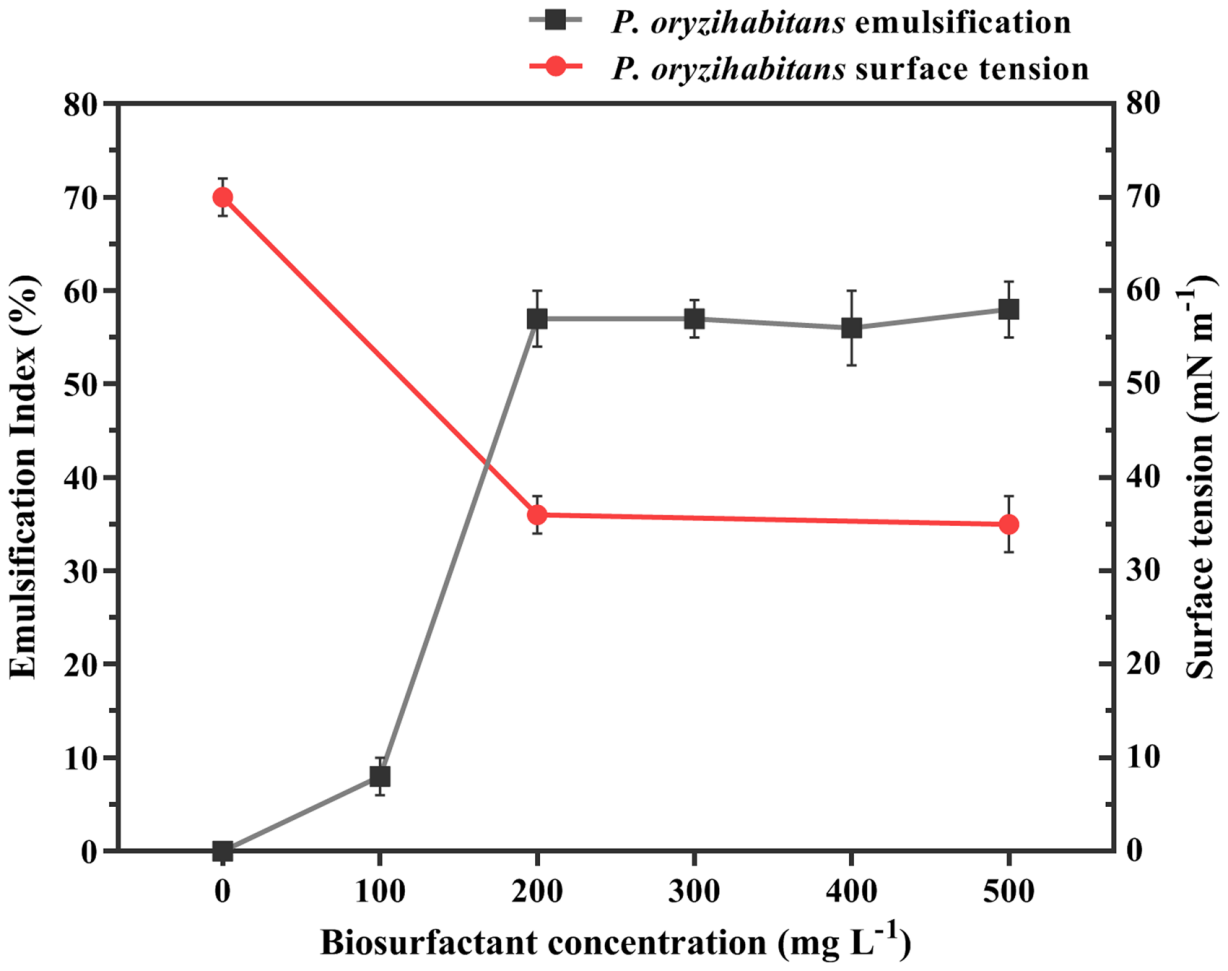
Emulsification index and surface tension as the function of the biosurfactant concentration (the biosurfactant isolated from *P. oryzihabitans* NC392 culture after the biodegradation process of crude oil at 15 g L^−1^). The experiment was performed in three replicates.

The tendency of surfactants to adsorb on surfaces due to their amphiphilic structure is one of their main characteristics (Santos et al., 2016; Sarubbo et al., 2022). When the concentration of surfactant increases, the surface tension goes down until it reaches a certain point and then stays the same because the surface is already saturated with surfactant molecules. The concentration needed for this to happen is called the critical micelle concentration (CMC) (Haba et al., 2003; Abdel-Mawgoud et al., 2008). In this study, the CMC was measured by determining the dependency of the emulsification index and surface tension on the concentration of the isolated biosurfactant in the aqueous system. The results showed that, based on the results obtained from E24 and surface tension measurement, the outcomes were linearized from the concentration of 200 mg L^−1^, and there was no noticeable decrease or increase. The concentration of 200 mg L^−1^ of extracted biosurfactant indicates the critical micelle concentration, which reduced the surface tension from 70 to 34 mN m^−1^ (Figure 2). Previous reported different values of the critical concentration of micelles, which may be due to the type of bacteria, different conditions of biosurfactant production, the nature of the dissolved solvent, and also the purity of biosurfactant preparation (Fox and Bala, 2000; Carrillo et al., 2003; Yin et al., 2009).

### 3.2 Chemical identification of the isolated biosurfactant

**Figure 3:**
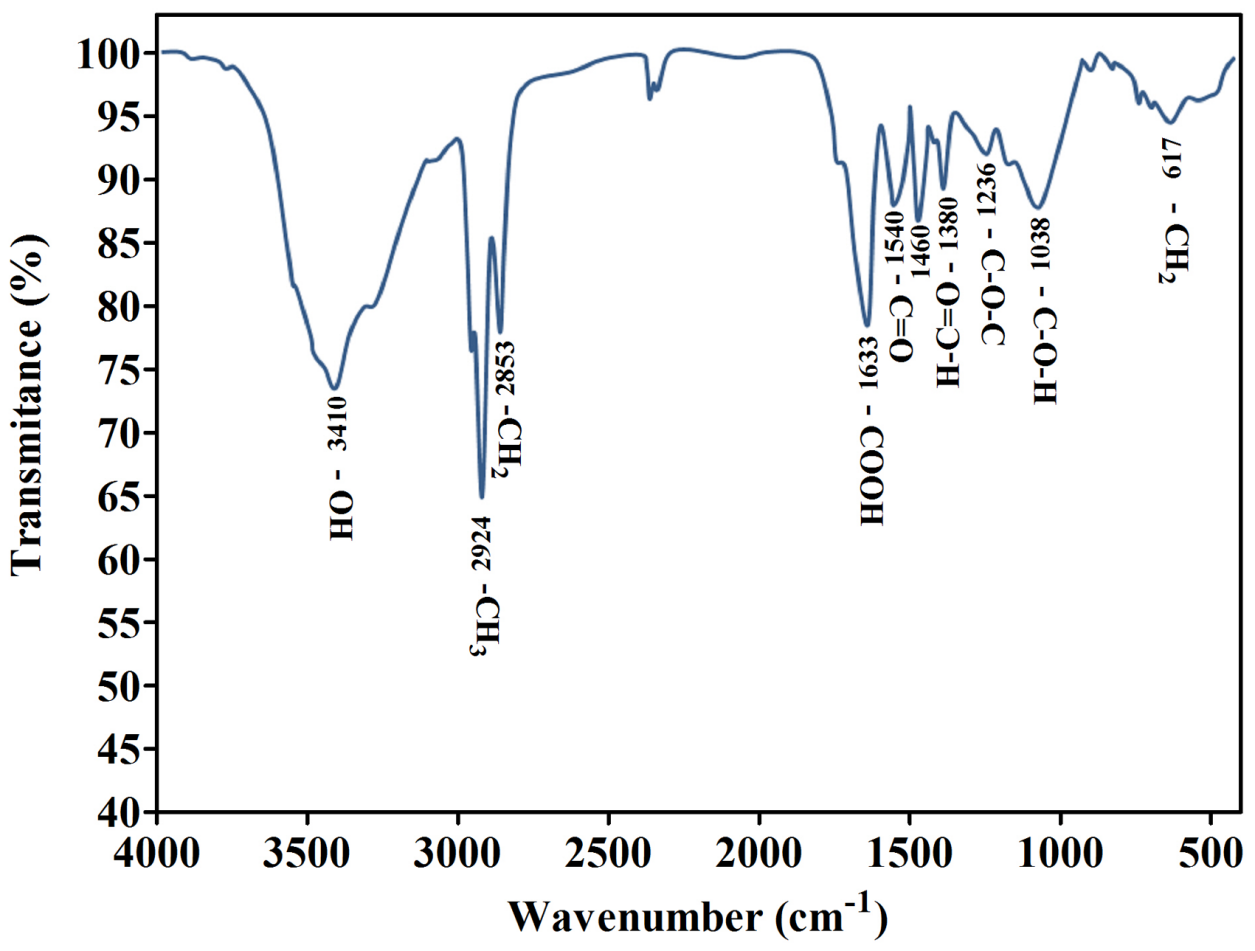
Fourier transform infrared spectrum obtained from the biosurfactant produced by *P. oryzihabitans.*

Numerous studies have described the isolation and synthesis of biosurfactants by various *Pseudomonas* species. The homologs of *P. aeruginosa*, a rhamnolipid producer, vary in the number of rhamnose molecules, alkyl chain length, and composition (Aparna et al., 2012). According to earlier research, the differences between functional groups found in the biosurfactant data were noticed and examined (Patowary et al., 2017). By using FTIR, the molecular makeup of the biosurfactant produced by *P. oryzihabitans* was assessed (Figure 3). The presence of OH bonds is indicated by the distinctive band at 3,410 cm^−1^. Around 2,924 cm^−1^ of absorption is attributed to the symmetric stretch (CH) of the aliphatic chains’ CH_2_ and CH_3_ groups. The absorption signal at 1,633 cm^−1^ (CO bond in COOH) shows the presence of ester carbonyl groups. Although other groups also absorb in this area, the band at 1,236 cm^−1^ further demonstrated the ester carbonyl group, which corresponds to CO deformation vibrations (Figure 3). The glycosidic bond (C-O-C) in the molecule is represented by the absorption band at 1,038 cm^−1^. The existence of the CH_2_ group is indicated by the absorption peak at 617 cm^−1^. According to the information from the corresponding wave numbers above, this biosurfactant’s chemical structure is the same as that of rhamnolipids that have been previously described. These rhamnose rings are connected to lengthy hydrocarbon chains (Pornsunthorntawee et al., 2008).

**Figure 4:**
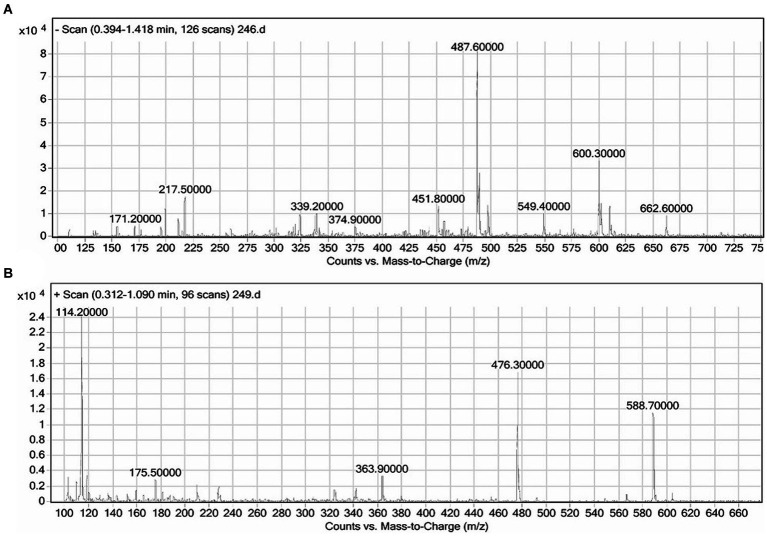
The results of the LC–MS analysis of the biosurfactants produced by *P. oryzihabitans* at different ionization modes: **(A)** Negative and **(B)** positive ionization mode.

The crude biosurfactant was subjected to negative and positive ion mode LC–MS analysis to identify its structural components. In the column purified biosurfactant, five rhamnolipid congeners were identified (Figure 4) after comparing the LC–MS data acquired for the biosurfactant with those published in earlier literature (Abdel-Mawgoud et al., 2010; Pantazaki et al., 2011). A methylated monorhamnolipid congener matching to [Rha-(C10-C10:1)-CH_3_] was identified in the spectra at m/z 588. As well as prominent peaks at m/z 363 and 476 were found, which corresponded to [M + H]^+^ ion of [Rha-C12:2] and [Rha-C8-C8], respectively. The spectra also showed a correlation between the molecular [M + H]^+^ ion and a sodium adduct [M + Na]^+^ ion of the same di-rhamnolipid congener [Rha-Rha-C10-C8] at m/z 600 and 662. Additionally, a di-rhamnolipid congener with m/z 549 was detected in the spectra, which corresponds to the deprotonated molecule [M-H]^−^ of [Rha-Rha-C8-C8]. As a result, it can be inferred that the produced biosurfactant by the *P. oryzyhabitans* strain NC392, using crude oil hydrocarbons as the carbon source, consists of a combination of both di and mono-rhamnolipid (Figure 4). The research that is currently accessible indicates that under natural circumstances, different strains of *P. aeruginosa* are known to produce a variety of distinct mono-and di-rhamnolipid congeners (Patowary et al., 2016). According to published research, *P. aeruginosa* produces a variety of mono-and rhamnolipid congeners of rhamnolipid biosurfactants that, through their microbiological and physicochemical effects on contaminant availability, essentially enhance the biodegradation of hydrocarbon contaminants such as pyrene, phenanthrene, hexadecane, and other crude oil components (Hwang and Cutright, 2002; Noordman et al., 2002; Das et al., 2014). *Pseudomonas* produces rhamnolipids through several steps (Abdel-Mawgoud et al., 2014): The precursors for rhamnolipid production include the sugar (dTDP-l-rhamnose) and hydrophobic compounds like 3-(3-hydroxyalkanoyloxy) alkanoic acid (HAA). D-glucose is used to synthesize the sugar moiety, while the fatty acid synthesis pathway (i.e., oxidation pathway) is used to synthesize the hydrophobic moiety. Most bacteria possess the enzymes needed to synthesize the precursors in rhamnolipid biosynthesis; however, the enzymes involved in the synthesis of HAA (RhiA), mono-rhamnolipids (RhiB), and di-rhamnolipids (RhiC) are found almost exclusively in *Pseudomonas* sp. (Dobler et al., 2016). The synthesis of rhamnolipids and their precursors occurs upon quorum sensing (QS) induction in the bacterial communication system. Regulating rhamnolipid production in *P. aeruginosa* involves three QS systems, including las, rhl, and pqs systems (Dusane et al., 2010). Nevertheless, the presence of water-insoluble substrates like hydrocarbons in the medium can also lead to the synthesis of rhamnolipids (Matvyeyeva et al., 2014; Chong and Li, 2017).

**Figure 5:**
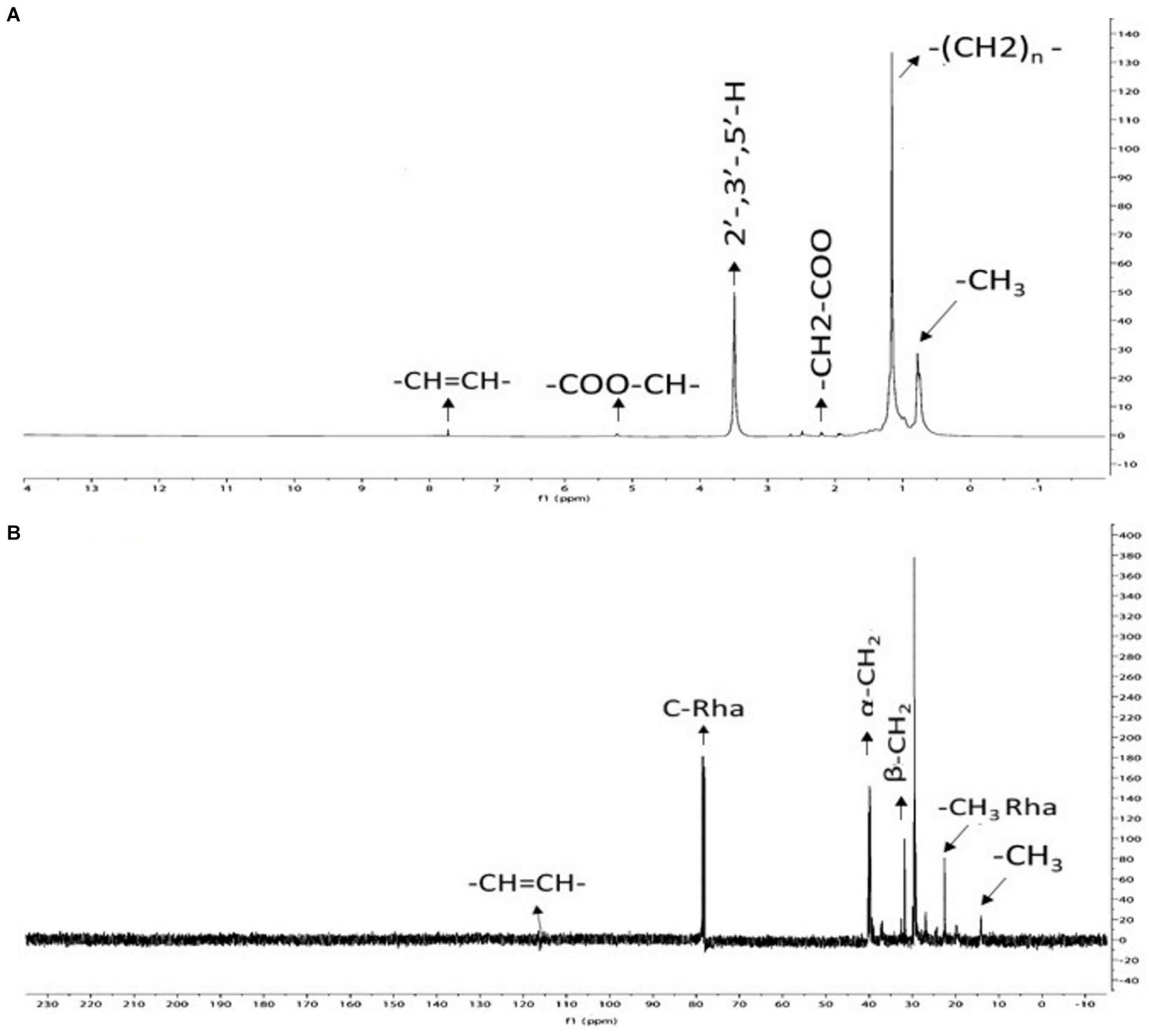
The results of nuclear magnetic resonance (NMR) analysis on the biosurfactant produced by *P. oryzihabitans*: **(A)** ^13^C-NMR, and **(B)** ^1^H-NMR.

Using ^13^C and ^1^H NMR spectroscopy, the structural properties of *P. oryzihabitans* strain NC392 biosurfactant was studied, and the results are presented in Figure 5. The biosurfactant’s methyl group (-CH_3_), which corresponds to the sugar moiety, is present as indicated by the peaks at 0.86–0.92 ppm in Figure 5A. Methyl groups have been reported to have the capability to make biosurfactants more hydrophobic (Pereira et al., 2013). Also, lipid signals present in rhamnolipid consisting of -CH_2_-bonds are detected at 1.16–1.35 ppm. The peaks at 2.20–2.32 ppm and 5.24 ppm, respectively, corresponded to CH_2_–COO and COO-CH groups. The Ar–H groups are associated with the peak 3.6 ppm. To summarize, the ^1^H NMR spectrum verifies the presence of carboxylic, methyl, hexose sugar, aromatic moieties in the biosurfactant, and it is in line with previous rhamnolipid obtained from other sources (Lotfabad et al., 2010; Pereira et al., 2013). The appearance of identifying signals for rhamnolipid at 13.7 ppm (CH_3_), 22.5–32.7 ppm (-CH_2_-), and 78.8–79.3 ppm (Rha-carbon) were observed in the ^13^C NMR spectrum (Figure 5B).

### 3.3 Stability analysis on the isolated biosurfactants

**Figure 6:**
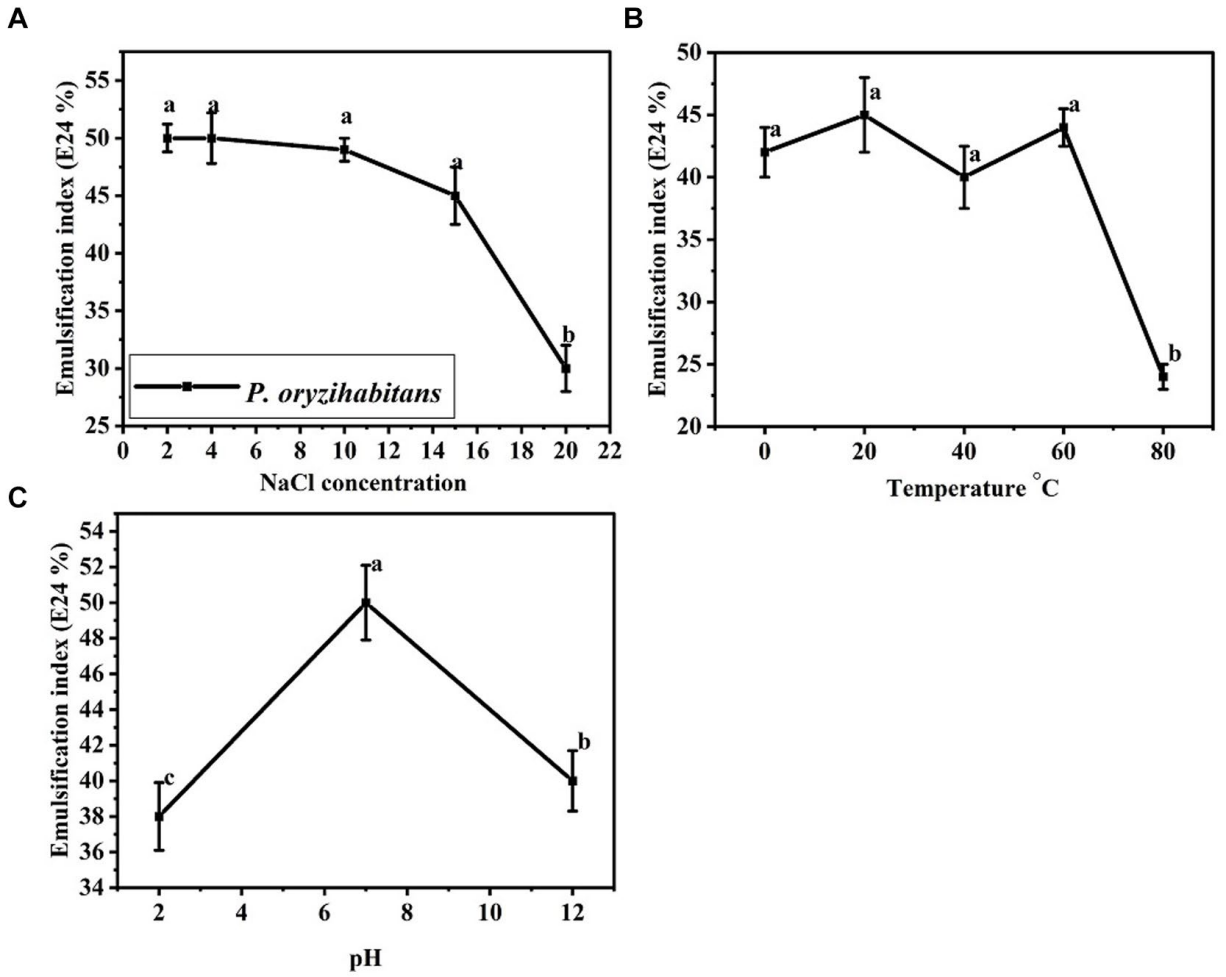
Effects of different parameters on the emulsification index of the biosurfactant produced by *P. oryzihabitans* NC392: **(A)** NaCl concentration, **(B)** temperature, and **(C)** pH. Bars on the columns indicate the standard error of the mean. The difference between means with common letters is not statistically significant.

Considering that petroleum contamination sometimes occurs in extreme environmental areas, the efficiency of biosurfactants in different fields depends on their stability in different environmental conditions (Almansoory et al., 2019; Sánchez, 2022). The effect of parameters such as pH, temperature, and salinity concentration on the activity of biosurfactants has been investigated in previous studies for different microorganisms producing biosurfactants (Aparna et al., 2012; Khopade et al., 2012a; Almansoory et al., 2019). In the present study, we also investigated the stability of the produced biosurfactant under different conditions. The results are shown in Figure 6. The biosurfactant produced maintained its stability and tolerance in the 0 to 60°C temperature range. The stability of biosurfactants in a wide temperature range can increase the applications of the produced biosurfactant (Jahan et al., 2020). The activity of biosurfactants is usually the highest at neutral pH (Lima and Alegre, 2009). In our study, the produced biosurfactant showed the highest emulsification rate at neutral pH. The salinity concentration of up to 15% NaCl did not have much effect on the emulsification index of the produced biosurfactant, but the concentration of more than 15% strongly reduced the emulsification rate. This level of stability at a high concentration of salinity (15%) shows high stability. In total, the biosurfactant produced by *P. oryzihabitans* using crude oil as sole of carbon was a stable biosurfactant with neutral acidity and a temperature range of 0 to 60°C, which could tolerate a high salinity concentration (15%). This level of stability of this biosurfactant makes it a potential alternative to increase the bioremediation of oil contamination in areas with extreme conditions.

## 4 Conclusion

This work showed the ability of *Pseudomonas oryzihabitans* NC392 cells to produce rhamnolipid molecules during the biodegradation process of crude oil hydrocarbons. The biosurfactants at a concentration equal to 200 mg L^−1^ reach the critical micelle concentration with a surface tension reduction from 68 to 34 mN m^−1^. According to FTIR, NMR, and LC–MS analyses, the produced biosurfactants were identified as the di-rhamnolipid and mono-rhamnolipid types. The emulsification capacity of the biosurfactant was stable under a wide range of temperatures, NaCl concentration, and pH, which indicates the potential application of the biosurfactants in bioremediation studies as eco-friendly and biodegradable products and its stability makes it optimal for areas with extreme conditions.

## Data Availability

The original contributions presented in the study are included in the article/supplementary material, further inquiries can be directed to the corresponding author.
